# Supplementary material for: Diagnostic Blood Biomarkers for Acute Pulmonary Embolism: A Systematic Review
Source: Diagnostics (Basel). 2023 Jul 6;13(13):2301. doi: 10.3390/diagnostics13132301 (PMC10340158; doi:10.3390/diagnostics13132301)
Supplement: Supplementary file 1 [file diagnostics-13-02301-s001.zip › diagnostics-2428428-supplementary.pdf]

Supplementary materials

Tables

Table S1. Medline search strategy.

**Database: Ovid MEDLINE(R) and In-Process, In-Data-Review & Other Non-Indexed Citations and Daily** **1946 to January 18, 2023**			
#	Searches	Results (hits)	Date
1	exp pulmonary embolism/	42897	18.01.23
2	PE.ti,ab,kw	61233	18.01.23
3	PTE.ti,ab,kw	2932	18.01.23
4	Lung adj3 embol*.ti,ab,kw	1231	18.01.23
5	Lung adj3 thromb*.ti,ab,kw	1033	18.01.23
6	Lung adj3 clot*.ti,ab,kw	61	18.01.23
7	Lung adj3 infarc*.ti,ab,kw	428	18.01.23
8	Pulmonary adj3 embol*.ti,ab,kw	46730	18.01.23
9	Pulmonary adj3 thromb*.ti,ab,kw	18864	18.01.23
10	Pulmonary adj3 clot*.ti,ab,kw	257	18.01.23
11	Pulmonary adj3 infarct*.ti,ab,kw	2520	18.01.23
12	Or/1-11	123649	18.01.23
13	exp biomarker/an	144713	18.01.23
14	exp biomarker/bl	200280	18.01.23
15	exp biomarker/ge	104820	18.01.23
16	exp biomarker/im	54809	18.01.23
17	exp biomarker/me	212343	18.01.23
18	"Diagnostic techniques and procedures"/	3680	18.01.23
19	Clinical laboratory techniques/	23783	18.01.23
20	Clinical chemistry tests/	1584	18.01.23
21	Hematologic test/	10079	18.01.23
22	Molecular diagnostic techniques/	13650	18.01.23
23	Biomarker*.ti,ab,kw	386345	18.01.23
24	Biologic* adj3 marker*.ti,ab,kw	13639	18.01.23
25	Biologic* adj3 indicator*.ti,ab,kw	3473	18.01.23
26	Biologic* adj3 test*.ti,ab,kw	9864	18.01.23
27	Biochemic* adj3 marker*.ti,ab,kw	18765	18.01.23
28	Biochemic* adj3 indicator*.ti,ab,kw	3346	18.01.23
29	Biochemic* adj3 test*.ti,ab,kw	15604	18.01.23
30	Laboratory adj3 marker*.ti,ab,kw	3466	18.01.23
31	Laboratory adj3 indicator*.ti,ab,kw	1451	18.01.23
32	Laboratory adj3 test*.ti,ab,kw	64407	18.01.23
33	Molecular adj3 marker*.ti,ab,kw	34477	18.01.23
34	Molecular adj3 indicator*.ti,ab,kw	709	18.01.23
35	Molecular adj3 test*.ti,ab,kw	18079	18.01.23
36	Or/13-35	1094927	18.01.23
37	12 and 36	5900	18.01.23
38	limit 37 to (english language and humans and yr="1995 -Current")	3900	18.01.23

Table S2. Embase search strategy.

Database: Ovid EMBASE Classic+Embase 1947 to January 18, 2023			
#	Searches	Results (hits)	Date
1	Exp *Lung embolism/	41377	18.01.23
2	PE.ti,ab,kw	78358	18.01.23
3	PTE.ti,ab,kw	4615	18.01.23
4	Lung adj3 embol*.ti,ab,kw	2238	18.01.23
5	Lung adj3 thromb*.ti,ab,kw	1739	18.01.23
6	Lung adj3 clot*.ti,ab,kw	108	18.01.23
7	Lung adj3 infarc*.ti,ab,kw	715	18.01.23
8	Pulmonary adj3 embol*.ti,ab,kw	78440	18.01.23
9	Pulmonary adj3 thromb*.ti,ab,kw	30239	18.01.23
10	Pulmonary adj3 clot*.ti,ab,kw	454	18.01.23
11	Pulmonary adj3 infarct*.ti,ab,kw	4539	18.01.23
12	Or/1-11	164067	18.01.23
13	Biochemical marker/	17436	18.01.23
14	*Biological marker/	112869	18.01.23
15	Molecular marker/	16756	18.01.23
16	Diagnostic test/	87231	18.01.23
17	Diagnostic procedure/	99024	18.01.23
18	Biochemical analysis/	40360	18.01.23
19	Exp blood analysis/	199186	18.01.23
20	Biomarker*.ti,ab,kw	577869	18.01.23
21	Biologic* adj3 marker*.ti,ab,kw	19057	18.01.23
22	Biologic* adj3 indicator*.ti,ab,kw	4472	18.01.23
23	Biologic* adj3 test*.ti,ab,kw	14076	18.01.23
24	Biochemic* adj3 marker*.ti,ab,kw	26849	18.01.23
25	Biochemic* adj3 indicator*.ti,ab,kw	4379	18.01.23
26	Biochemic* adj3 test*.ti,ab,kw	23842	18.01.23
27	Laboratory adj3 marker*.ti,ab,kw	5871	18.01.23
28	Laboratory adj3 indicator*.ti,ab,kw	2101	18.01.23
29	Laboratory adj3 test*.ti,ab,kw	103642	18.01.23
30	Molecular adj3 marker*.ti,ab,kw	43604	18.01.23
31	Molecular adj3 indicator*.ti,ab,kw	905	18.01.23
32	Molecular adj3 test*.ti,ab,kw	28046	18.01.23
33	Or/13-32	1239547	18.01.23
34	12 and 33	9177	18.01.23
35	limit 34 to (human and english language and yr="1995 -Current" and (article or article in press or books or chapter or conference paper or "conference review" or editorial or erratum or letter or note or "preprint (unpublished, non-peer reviewed)" or "review" or short survey or tombstone))	4548	18.01.23

Table S3. The reason for exclusion of studies during full text eligibility assessment.

Study (Surname year)	Main reason	Comment
**1. Acat 2020**	Two-gate case-control study	
**2. Alqudah 2017**	Two-gate case-control study	
**3. Aykal 2015**	Two-gate case-control study	
**4. Berk 2013**	Insufficient reporting	Not conducted diagnostic statistical analyses and not chosen a cut-off value.
**5. Dawood 2014**	Two-gate case-control study	
**6. Farm 2020**	Disease of interest was not acute PE	Authors did not report separate data for pulmonary embolism patients.
**7. Flores 2016**	Duplicate publication	A study with the same index test and study population reporting the same diagnostic results by these authors was published by another journal two years earlier, which is included in the systematic review.
**8. Ghahnavieh 2019**	Insufficient reporting	
**9. Gul 2016**	Unacceptable reference standard	CT with and without angiography were accepted as reference standard. In addition, it was impossible to extract or calculate data to 2x2 contingency table.
**10. Han 2021**	Two-gate case-control study	
**11. Hogg 2012**	Insufficient reporting	Several aspects with this study which were unclear, an email has been sent for clarification. The study has not reported a cut-off for the biomarker.
**12. In 2015**	Two-gate case-control study	
**13. Karatas 2018**	Two-gate case-control study	
**14. Kuluozturk 2019**	Two-gate case-control study	
**15. Metafratzi 2006**	Insufficient reporting	
**16. Nordenholz 2008**	Unacceptable reference standard	D-dimer accepted as reference standard.
**17. Ozturk 2016**	Insufficient reporting	
**18. Pomero 2013**	Insufficient reporting	
**19. Rodger 2000**	Ineligible study population	Inpatients included.
**20. Sainaghi 2009**	Unacceptable reference standard	The investigators used both lung ventilation perfusion scan and CT pulmonary angiography as reference standard. The proportion of patients of which received a ventilation perfusion scan was not reported, and it was not reported a justification for the usage of the procedure.
**21. Singer 2009**	Unacceptable reference standard	CT pulmonary angiography, lung ventilation perfusion scan and pulmonary angiography were accepted as reference standards. The proportion of patients receiving the different reference standards

		were not reported, and it was not reported a justification for the usage of ventilation perfusion scan and pulmonary angiography.
**22. Steeghs 2005**	Unacceptable reference standard	Forty percent of the patients received lung ventilation perfusion scan as reference standard.
**23. Wang 2018**	Two-gate case-control study	
**24. Wexels 2016**	Insufficient reporting	
**25. Yin 2009**	Two-gate case-control study	
**26. Zhang 2018**	Two-gate case-control study	
**27. Zhou 2021**	Two-gate case-control study	
**28. Bos 1999**	Two-gate case-control study	
**29. El-Habashy 2014**	Insufficient reporting	
**30. Gutte 2011**	Ineligible study population	Inpatients included.
**31. Heerink 2021**	Disease of interest was not acute PE	VTE disease of interest, not reported separate diagnostic data for pulmonary embolism.
**32. Heining 2016**	Insufficient reporting	
**33. Liu 2018**	Two-gate case-control study	
**34. Lupi-Herrera 2018**	Ineligible study population	
**35. Ozmen 2020**	Two-gate case-control study	
**36. Ozyurt 2020**	Two-gate case-control study	
**37. Reber 1999**	Unacceptable reference standard	
**38. Talay 2014**	Two-gate case-control study	
**39. Xiao 2011**	Two-gate case-control study	
**40. Yolcu 2014**	Two-gate case-control study	
**41. Zhou 2015**	Two-gate case-control study	
**42. Bakirci 2015**	Two-gate case-control study	
**43. Bozorgmehr 2019**	Ineligible study population	Inpatients included
**44. Dirican 2017**	Two-gate case-control study	
**45. Duman 2019**	Two-gate case-control study	
**46. Farah 2020**	Disease of interest was not acute PE	VTE disease of interest, not reported separate diagnostic data for pulmonary embolism.
**47. Kaya 2012**	Two-gate case-control study	
**48. Kessler 2016**	Two-gate case-control study	
**49. Kilinic 2012**	Unacceptable reference standard	Lung ventilation perfusion scan was the only reference standard.
**50. Lu 2022**	Two-gate case-control study	
**51. Mitchell 2008**	Insufficient reporting	
**52. Morris 2003**	Two-gate case-control study	
**53. Schroeder 2003**	Insufficient reporting	
**54. Turedi 2007**	Two-gate case-control study	
**55. Usul 2020**	Two-gate case-control study	
**56. Yilmaz 2016**	Ineligible study population	Inpatients included.
**57. Bonfanti 2021**	Insufficient reporting	Not reported sensitivity and specificity of the ROC-analysis on troponin I.
**58. Dirican 2016**	Insufficient reporting	Authors included 25 healthy controls in the group free of acute pulmonary embolism in the receiver operating curve-analysis of the index test.

**59. Melanson 2006**	Unacceptable reference standard	The group of patients free of acute pulmonary embolism did not receive a CT pulmonary angiography.
**60. Smith 2022**	Two-gate case-control study	
**61. Kara 2022**	Insufficient reporting	
	**Articles from references**	
**1. Crop 2013**	Insufficient reporting	Cut-off value, sensitivity and specificity for C-reactive protein were not reported.
**2. Ginsberg 1996**	Unacceptable reference standard	Only lung ventilation perfusion scan was used as reference standard.
**3. Wada 2008**	Publication type	Narrative review.
**4. Turedi 2008**	Two-gate case-control study	
**5. Stein 1996**	Unacceptable reference standard	The investigators used both lung ventilation perfusion scan and CT pulmonary angiography as reference standard. The proportion of patients of which received a ventilation perfusion scan was not reported, and it was not reported a justification for the usage of the procedure.
**6. Riva 2018**	Ineligible study population	Patients were not suspected to have acute pulmonary embolism.
**7. LaCapra 2000**	Unacceptable reference standard	Lung ventilation perfusion scan and pulmonary angiography were the only reference standards.
**8. Varol 2011**	Two-gate case-control study	
**9. Moharamzadeh 2019**	Insufficient reporting	
**10. Aujesky 2003**	Unacceptable reference standard	Not all patients received a CT pulmonary angiography, but all patients received a compression ultrasound procedure. The use of compression ultrasound was not justified.
**11. Hogg 2011**	Ineligible study population	Inpatients included.

Abbreviations:
CT, computed tomography; ROC, receiver operating curve; VTE, venous thromboembolism.

Table S4 . The included studies' reported list of exclusion criteria.

**Study (first author and publication year)**	**Exclusion criteria**
Celik et al., 2015 (1)	Patients with active or chronic inflammatory or autoimmune diseases; inflammatory rheumatic disease; anemia; clinical evidence of active infection; active cancer; any hematological diseases; recent blood transfusion; chronic renal disease; and history of chronic obstructive pulmonary disease
Çevik et al., 2018 (2)	Congestive heart failure, hematological or oncologic disease, chronic infection, vasculitis, coronary artery disease, peripheral arterial disease, pregnancy, liver and kidney failure, and previous cerebrovascular disease
Ebrahimi et al., 2022 (3)	Not agreeing to participate in the study, pregnancy, renal failure, treatment with anticoagulants, myocardial infarction, need for intubation, myocarditis, massive embolism, hypertrophic cardiomyopathy, and negative D-dimer
Flores et al., 2014 (4)	Patients younger than 18 years, pregnant patients, patients already on therapeutic anticoagulation, logistic reasons (unavailability of radiological procedures)
Huang et al., 2015 (5)	Acute coronary syndrome, haematological disorders such as thrombocytosis and idiopathic thrombocytopenic purpura, severe hepatic and renal diseases, chronic pulmonary hypertension, diabetes mellitus, malignancy, and use of anticoagulation therapy
Kalkan et al., 2016 (6)	Sepsis, lung neoplasms, end-stage renal failure requiring hemodialysis treatment, acute coronary syndromes, acute cerebrovascular disease, acute or chronic aortic dissection, decompensated heart failure, surgery or bed rest within the past 30 days, prior PE or deep venous thrombosis, severe chronic obstructive lung disease (FEV1<50%), pulmonary hypertension, acute or chronic infectious diseases, acute or chronic inflammatory diseases such as acute myocarditis and/or pericarditis, chronic constrictive pericarditis, rheumatoid arthritis, systemic lupus erythematosus, vasculitis

Table S5. Review specific, standardized form for risk of bias assessment based on The Quality assessment of diagnostic accuracy studies 2 (QUADAS-2) (7).

QUADAS-2 review-specific standardized form	
State the review question. Describe the index test, reference standard and population.	
Draw or insert a flowchart of the patient flow in the primary study.	
Description, signalling questions (SQ) and overall evaluation of risk of bias domain	Rating criteria
Domain 1: Patient selection	
Description	Describe methods of patient enrollment.
SQ1: Was a consecutive or random sample of patients enrolled?	**Yes:** It is stated that the study sample was consecutive or random.<br>**No:** It is stated that the study sample was not consecutive or random or it is stated that convenience sampling was conducted.<br>**Unclear:** The method of sampling is ambiguous or not reported at all.
SQ2: Did the study avoid inappropriate exclusions?	**Yes:** The included patients in the study resemble to a large extent the population which would have received the index test in clinical practice as an outpatient or patient admitted to an emergency department (target population).<br>**No:** The exclusion criteria reported in the study increases the risk selection of patients, indicated by differences in demographics and spectrum of patients in study population compared to target population in clinical practice.<br>**Unclear:** The inclusion and exclusion criteria are not reported.<br><br><u>Review specific considerations:</u> Exclusion criteria which are related to common differential diagnosis of pulmonary embolism (e.g., coronary artery diseases, lower acute respiratory infections, chronic obstructive pulmonary disease, exacerbations, anxiety attacks etc.), or very prevalent diseases which could alter the demographics of the study population to such an extent that the sample does not longer resemble the population in clinical practice (e.g., all cancer patients, all patients which hematological diseases, coronary diseases, all which reduced kidney function etc.) – are considered inappropriate exclusion criteria.
Overall evaluation of risk of bias in domain 1:	Is the risk that the selection of patients in this study could have introduced bias:<br>**Low, high, or unclear?**<br><br><u>Review specific considerations:</u> The exclusion criteria of this systematic review have ensured that all included studies have avoided a case-control design. This fact must be taken in account in addition to the SQ in the overall evaluation of risk of bias. Two-gate case-control design often leads to an overestimation of diagnostic measurements.
Domain 2: Index test	
Description	Describe the index test and how it was conducted and interpreted.

SQ1: Were the index test results interpreted without knowledge of the results of the reference standard?	**Yes:** It is stated that the personnel interpreting index test was blinded to the results of the reference standard.<br>**No:** It is stated that the personnel interpreting index test was not blinded to the results of the reference standard. Or it is reported that same person was the one to interpret both index test(s) and reference standard.<br>**Unclear:** If it is not reported who interpreted the index test.<br><br><u>Review specific considerations:</u> Blood biomarkers measured in hospitals are often analyzed by the hospital laboratory personnel, which we assume do not have the access to (or have the interest to know) the radiological procedures test results. Therefore, we assume that for most of biomarkers identified by this review, the persons interpreting the index test is blinded to the reference standard results. Especially if the biomarkers are a part of the routine blood tests in the hospitals.
SQ2: If a threshold was used, was it pre-specified?	**Yes:** The study reports a pre-specified threshold for the biomarker (index test).<br>**No:** The study has not reported a pre-specified threshold and has used ROC-analysis to find the optimal cut-off.<br>**Unclear:** Ambiguous reporting of the cut-off which makes it unclear if was derived from.<br><br><u>Review specific considerations:</u> The risk of bias in the index domain should not be influenced by this SQ if the biomarker investigated does not have a conventional cut-off for pulmonary embolism. This review's aim is to identify non-established biomarkers for pulmonary embolism, and to investigate their clinical, diagnostic utility. Therefore, most of the biomarkers identified through this review will not have conventional cut-offs for pulmonary embolism.
SQ3: Was treatment withheld until the index test was performed?	This question was added to the QUADAS-2 tool.<br>**Yes:** It is stated that treatment of acute pulmonary embolism was withheld until blood samples for the index test were drawn.<br>**No:** It is stated that some or all patients received antithrombotic treatment for acute pulmonary embolism before blood samples for the index test were drawn.<br>**Unclear:** It was not reported whether treatment was initiated or not before blood samples were drawn.<br><br><u>Review specific considerations:</u> If the study reported that the blood samples were taken at admission or that the blood samples were a part of the routine blood tests taken at the emergency department, we considered the probability that the patients has received anticoagulant treatment, thrombolysis, or embolectomy as low (yes on SQ3).
Overall evaluation of risk of bias in domain 2:	Is the risk that the conduct or interpretation of the index test have introduced bias:<br>**Low, high, or unclear?**<br><br><u>Review specific considerations:</u> Some studies report diagnostic data on more than one index test. If the conduct or interpretation of these tests differ, the index test(s) with the largest diagnostic potential to correctly classify (highest validation measurements and fewest signs of imprecision) pulmonary embolism patients should influence this domain the most.
Domain 3 Reference standard	
Description	Describe the reference standard and how it was conducted and interpreted.

SQ1: Were the reference standard results interpreted without knowledge of the results of the index test?	**Yes:** It is stated that the personnel interpreting reference standard was blinded to the results of the index test(s).<br>**No:** It is stated that the personnel interpreting reference standard was not blinded to the results of the index test(s). Or it is reported that same person was the one to interpret both reference standard and index test(s).<br>**Unclear:** If it is not reported who interpreted the reference standard.<br><br>Review specific considerations: If the study reports that radiologists were the ones to interpret the findings of the reference standard; we consider the risk that the radiologist are influenced by the blood sample results as being low especially if the study data is collected retrospectively (since they did not know that their radiological judgements were going to influence the study results).
SQ2: Was treatment withheld until the reference standard was performed?	This question was added to the QUADAS-2 tool.<br>**Yes:** It is stated that treatment of acute pulmonary embolism was withheld until the reference standard was conducted.<br>**No:** It is stated that some or all patients received antithrombotic treatment for acute pulmonary embolism before the reference standard was conducted.<br>**Unclear:** It was not reported whether treatment was initiated or not the reference standard was conducted.<br><br>Review specific considerations: We consider the chance that anticoagulation treatment causes the pulmonary artery embolus/thrombus to completely vanish, and thus, resulting in a false negative test result of the reference standard as low. However, if the patients received thrombolysis or embolectomy, we consider the chance that treatment could influence the result of the reference standard as higher.
Overall evaluation of risk of bias in domain 3:	Is the risk that reference standard, its conduct, or its interpretation have introduced bias:<br>**Low, high, or unclear?**<br><br>Review specific considerations: If the study uses other reference standards that has low a diagnostic accuracy for pulmonary embolism such as compression ultrasound, even though the usage may be justified, the study should be evaluated to have a high risk of bias in this domain.
Domain 4 Flow and timing	
Description	Use the flow chart inserted or drawn of the patient flow in the primary study.<br>1) Describe any patients who did not receive the index test(s) and/or reference standard or who were excluded from the 2x2 contingency table (refer to flow diagram).<br>2) Describe the time interval and any interventions between index test(s) and reference standard.
SQ1: Was there an appropriate interval between index test(s) and reference standard?	**Yes:** The study has reported an appropriate interval between index test(s) and reference standard. The time interval should be as short as possible, ideally within first day of admission.<br>**No:** The study has reported an inappropriate interval between index test(s) and reference standard. If the time interval is reported to be over 48 hours, it is not appropriate.<br>**Unclear:** The interval between the tests is not reported or difficult to interpret.

SQ2: Did all patients receive a reference standard?	**Yes:** The number of patients receiving a reference standard matches the number of the study sample.<br>**No:** The number of patients receiving a reference standard does not match the number of the study sample.<br>**Unclear:** The flow of patients is not reported properly and makes it impossible to answer the question.
SQ3: Were all patients included in the analysis?	**Yes:** The study reports that all the included patients were included in the statistical analyses (2x2 contingency table and ROC-analysis).<br>**No:** The study reports in the text/tables directly or you discover (by calculating the reported diagnostic estimated) that not all included patients are included in the statistical analyses (contingency table and ROC-analysis).<br>**Unclear:** The flow of patients is not reported properly and makes it impossible to answer the question.<br><br>Review specific considerations: If the calculated diagnostic estimates based on the reported true/false positive/negative test results do not match the reported sensitivity, specificity, negative/ positive predictive value you should assume that some patients are not included in the investigators analyses. Send email to corresponding author for clarification. If there is no good reason for why the diagnostic estimates are impossible to replicate, this SQ should be answered "No".
Overall evaluation of risk of bias in domain 4:	Is the risk that the flow of patients could have introduced bias:<br>**Low, high, or unclear?**<br><br>Review specific considerations: We have removed the signal question "Did all patents received the same reference standard" since one of this systematic review's criteria for inclusion is: all patients in the study needed to have undergone a reference standard to be eligible for this review.
**Abbreviations**:<br>QUADAS, quality assessment of diagnostic accuracy studies; ROC, receiver operating curve; SQ, signaling questions.	

References:

1. Celik A, Ozcan IT, Gündes A, Topuz M, Pektas I, Yesil E, et al. Usefulness of admission hematologic parameters as diagnostic tools in acute pulmonary embolism. Kaohsiung J Med Sci. 2015;31(3):145-9. https://doi.org/10.1016/j.kjms.2014.12.004
2. Çevik İ, Narcı H, Dündar GA, Ayrık C, Babuş SB. Is there a diagnostic value for the platelet indices patients in pulmonary embolism? HKJEM. 2018;25(2):91-4. https://doi.org/10.1177/1024907917743489
3. Ebrahimi M, Arab MM, Zamani Moghadam H, Jalal Yazdi M, Rayat Doost E, Foroughian M. Risk Stratification of Pulmonary Thromboembolism using Brain Natriuretic Peptide and Troponin I; a Brief Report. Arch Acad Emerg Med. 2022;10(1):e8. https://doi.org/10.22037/aaem.v10i1.1453
4. Flores J, García-Avello Á, Alonso E, Ruíz A, Navarrete O, Álvarez C, et al. Tissue plasminogen activator as a novel diagnostic aid in acute pulmonary embolism. Vasa. 2014;43(6):450-8. https://doi.org/10.1024/0301-1526/a000392
5. Huang J, Chen Y, Cai Z, Chen P. Diagnostic value of platelet indexes for pulmonary embolism. Am J Emerg Med. 2015;33(6):760-3. https://doi.org/10.1016/j.ajem.2015.02.043
6. Kalkan AK, Ozturk D, Erturk M, Kalkan ME, Cakmak HA, Oner E, et al. The diagnostic value of serum copeptin levels in an acute pulmonary embolism. Cardiol J. 2016;23(1):42-50. https://doi.org/10.5603/CJ.a2015.0077
7. Whiting PF, Rutjes AW, Westwood ME, Mallett S, Deeks JJ, Reitsma JB, et al. QUADAS-2: a revised tool for the quality assessment of diagnostic accuracy studies. Ann Intern Med. 2011;155(8):529-36. https://doi.org/10.7326/0003-4819-155-8-201110180-00009
